# Supplementary material for: Removing physiological motion from intravital and clinical functional imaging data
Source: eLife. 2018 Jul 9;7:e35800. doi: 10.7554/eLife.35800 (PMC6037484; doi:10.7554/eLife.35800)
Supplement: Supplementary file 1. [file elife-35800-supp1.docx]

|  |  | Acquisition Parameters | | | | Realignment Parameters | | |
| --- | --- | --- | --- | --- | --- | --- | --- | --- |
| **Dataset** | **Fig** | Pixels  (x-y,z) | Line rate (Hz) | Bidirectional scan? | Frames | Realignment points | Smoothing  radius (px) | Realignment threshold |
| Crypts, Rac1 | 4A | 256 | 1400 | Yes | 735 | 5 | 2 | 0.8 |
| Crypts, Rac1 (ex vivo) | 4B | 512 | 1000 | No | 230 | 20 | 4 | - |
| Intrasplenic, Src | 5E | 512 | 1000 | No | 230 | 15 | 4 | - |
| Skin, autofluorescence | 6A | 256 | 256 | No | 10 | 10 | 2 | - |
| Skin, autofluorescence | 6B | 256,36 | 116 | No | 56 | 6 | 0 | - |
| Lymph node | 7 | 512,51 | 1000 | Yes | 10 | 10 | 4 | - |
